# Analysis of number needed to treat for droxidopa in patients with symptomatic neurogenic orthostatic hypotension

**DOI:** 10.1186/s12883-016-0665-5

**Published:** 2016-08-18

**Authors:** Clément François, Gerald J. Rowse, L. Arthur Hewitt, Pamela Vo, Robert A. Hauser

**Affiliations:** 1 Lundbeck LLC, 6 Parkway North, Deerfield, IL 60015 USA; 2 Parkinson’s Disease and Movement Disorders Center, University of South Florida, 4001 E Fletcher Ave, Tampa, FL 33596 USA

**Keywords:** Neurogenic orthostatic hypotension, Droxidopa, nOH treatment benefit, Number needed to treat, Number needed to harm, Risk reduction

## Abstract

**Background:**

Droxidopa is an orally active prodrug that significantly improved dizziness/lightheadedness measured using the Orthostatic Hypotension Symptom Assessment (OHSA) Item 1 in patients with neurogenic orthostatic hypotension (nOH) caused by primary autonomic failure (Parkinson disease, multiple system atrophy, and pure autonomic failure), dopamine β-hydroxylase deficiency, or nondiabetic autonomic neuropathy. The efficacy and safety of droxidopa were assessed by determining the number needed to treat (NNT) and the number needed to harm (NNH).

**Methods:**

Data collected in randomized, placebo-controlled clinical studies in adults with a clinical diagnosis of symptomatic nOH were pooled for efficacy and safety analyses. NNT and NNH were calculated as reciprocals of the risk difference (difference in event rates) for droxidopa versus placebo.

**Results:**

The NNT for droxidopa for improvement in OHSA Item 1 was <10. The NNH for adverse events (AEs) leading to discontinuation in the pooled studies was 81. The likelihood of being helped or harmed (LHH) calculated from pooled analysis of the NNT for ≥2 units of improvement in OHSA Item 1 score and the NNH for discontinuations due to AEs were 7.8, 8.8, 3.1, and 3.5 for weeks 1, 2, 4, and 8 after randomization, respectively.

**Conclusions:**

Droxidopa is efficacious for treatment of nOH, with an NNT below 10 and an acceptable tolerability profile with NNH ranging from 23 to 302 in the pooled analysis of frequently occurring AEs. Based on the LHH for the pooled analysis at week 1, droxidopa is 7.8 times more likely than placebo to show a clinical benefit than result in discontinuation because of an AE.

**Trial registrations:**

ClinicalTrials.gov identifiers: NCT00782340, first received October 29, 2008; NCT00633880, first received March 5, 2008; and NCT01176240, first received July 30, 2010.

## Background

Symptomatic neurogenic orthostatic hypotension (nOH) occurs in patients with neurodegenerative disorders because of insufficient release of norepinephrine following a postural change to a standing position [1]. Droxidopa is an orally active prodrug of norepinephrine that was approved in 2014 by the US Food and Drug Administration (FDA) for the treatment of orthostatic dizziness, lightheadedness, or the “feeling that you are about to black out” in adult patients with symptomatic nOH caused by primary autonomic failure (Parkinson disease [PD], multiple system atrophy [MSA], and pure autonomic failure), dopamine β-hydroxylase deficiency (DBHD), or nondiabetic autonomic neuropathy (NDAN) [2].

The efficacy and safety of droxidopa have been investigated in several multicenter phase 3 studies in patients with symptomatic nOH using a validated instrument, the Orthostatic Hypotension Questionnaire (OHQ), to evaluate efficacy [3–6]. The OHQ, a patient-reported assessment of the burden and severity of nOH, consists of 2 parts: the Orthostatic Hypotension Symptom Assessment (OHSA), a measurement of symptoms, and the Orthostatic Hypotension Daily Activity Scale (OHDAS), a measurement of symptom impact on the activities of daily living [7]. In Study NOH301, compared with placebo, droxidopa treatment was associated with statistically significant improvements in OHQ composite score, standing systolic blood pressure (SBP), and the cardinal symptom of nOH (dizziness/lightheadedness) as measured using the OHSA Item 1 score [3]. Also, in the analysis of Study NOH306B as well as the combined analysis of Study NOH306, statistically significant improvements in OHSA Item 1 scores were observed after 1 week of treatment with droxidopa compared with placebo. In Study NOH302, patients with nOH treated with droxidopa had improvements in nOH symptoms as shown by a statistically significant decrease in the OHQ composite score and numerically greater decreases in most individual item scores compared with placebo [5, 6]. In all studies, treatment with droxidopa was generally well tolerated; commonly reported adverse events (AEs) included headache, dizziness, nausea, fatigue, and hypertension [3–6].

Although statistically significant clinical trial results are an essential measure of the efficacy of a medical intervention, their clinical relevance for the individual patient may be difficult to assess. To help clinicians further understand the risks and benefits of a potential treatment, calculation of the number needed to treat (NNT) and the number needed to harm (NNH) has been suggested [8, 9]. NNT is defined as the average number of patients that would need to be treated for 1 additional patient to achieve a benefit from treatment [9]. The converse of the NNT is the NNH, which estimates the average number of patients that need to be treated to reveal 1 additional adverse outcome [9].

Because nOH is a relatively rare disorder, there is an incomplete understanding of specific benchmarks of OHQ/OHSA score changes and their clinical relevance for the individual patient with nOH. Dizziness/lightheadedness is the cardinal symptom of nOH; improvement of this symptom may represent the most clinically relevant benefit of treatment of the disorder. Thus, this endpoint, as assessed by OHSA Item 1 score changes for droxidopa compared with placebo during clinical trials, was chosen as the outcome for evaluation in this post hoc analysis of efficacy, expressed in terms of the NNT. The current analysis also includes an evaluation of the safety of droxidopa, expressed as the NNH, using AEs experienced by patients during the same trials.

## Methods

### Patient populations of pooled clinical studies

Data collected in phase 3, randomized, placebo-controlled clinical studies were pooled for efficacy and safety analyses. Efficacy data were integrated from Studies NOH301 (NCT00782340) [3], NOH302 (NCT00633880) [6], and NOH306 (NCT01176240) [4, 5] to determine the NNT. The NNH analysis was performed using safety data from the same 3 studies. All 3 studies have been previously described in detail, and brief descriptions of the study designs, inclusion/exclusion criteria, and assessments are outlined below.

Patients included in all 3 studies were adults ≥18 years of age with a clinical diagnosis of symptomatic nOH that was confirmed at screening by a decrease of ≥20 mm Hg in SBP or ≥10 mm Hg in diastolic blood pressure (DBP) within 3 min after standing from a supine position. Patients in Studies NOH301 and NOH302 had primary diagnoses of PD, MSA, pure autonomic failure, NDAN, or DBHD. Study NOH306 enrolled patients with symptomatic nOH associated with PD. Key exclusion criteria in all studies included current use of vasoconstricting agents or long-acting antihypertensive medications, or preexisting severe hypertension (SBP >180 mm Hg or DBP >110 mm Hg in the standing, sitting, or supine position) [3–6].

### Study designs

In Studies NOH301and NOH302, all patients entered a ≤14-day dose titration period with open-label droxidopa to identify the maximum tolerated dose that reduced nOH symptoms (≥1 unit improvement in OHSA Item 1) and increased standing SBP by ≥10 mm Hg. In Study NOH301, responders entered a 7-day wash-out period and then were randomized to double-blind treatment with the optimized dose of droxidopa or placebo [3]. In Study NOH302, responders during the open-label titration period continued open-label droxidopa treatment at the optimized dose for 7 days before randomization to double-blind administration of placebo (ie, treatment withdrawal) or droxidopa [6]. In Study NOH306, patients were randomized to receive double-blind placebo or droxidopa for the ≤14-day dose titration period, which was followed by 8 weeks of maintenance treatment at each patient’s optimized dose. For the titration phase in all studies, droxidopa treatment was initiated at 100 mg 3 times daily (TID) and increased in 100-mg TID increments until a response was obtained, the patient was asymptomatic, or a maximum dose of 600 mg TID was reached.

In each study, nOH symptoms were assessed using the patient-rated OHQ scores (overall composite and individual item scores for OHSA and OHDAS). OHSA Item 1 measured the severity of dizziness/lightheadedness on a scale of 0 (none) to 10 (worst possible) [3–6]. In Study NOH306, patients also recorded all falls in a daily diary. Fall-related injuries were prespecified AEs occurring on the day of or the day after a reported fall. The AEs considered to be fall related were identified by the following *Medical Dictionary for Regulatory Activities* preferred terms: arthralgia, back pain, conjunctival hemorrhage, contusion, excoriation, face edema, facial bone fracture, fall, fibula fracture, foot fracture, headache, injury, joint sprain, laceration, musculoskeletal chest pain, musculoskeletal pain, musculoskeletal stiffness, neck pain, noncardiac chest pain, pain, pain in extremity, skin laceration, skin lesion, tooth fracture, traumatic brain injury, and traumatic hematoma [4].

In 2 of the studies in our analysis (Studies NOH301 and NOH302), only patients who met the responder criteria during the open-label dose optimization period and continued to the double-blind treatment period were included. Because NNT/NNH determinations require a comparison group for calculation, 181 patients (of a total 444 patients; 40.7 %) who discontinued during the open-label titration period were excluded from our analyses. The most common investigator-reported reasons for discontinuation during the open-label period were treatment failure (74/444, 16.7 %) and AEs/blood pressure elevation (55/444, 12.4 %).

### Outcomes

The primary study outcomes for this NNT/NNH analysis are the OHSA Item 1 improvement by ≥2 units and ≥50 %. OHSA Item 1 was chosen as the primary outcome (as opposed to the composite OHQ score), because this single assessment evaluates the cardinal symptoms of nOH, dizziness and lightheadedness. In addition, OHSA Item 1 was the primary outcome supported by the FDA Study Endpoints and Labeling Development Division for the droxidopa pivotal trials.

Because of the different periods of randomized treatment in the 3 droxidopa trials, separate NNTs were calculated based on duration of exposure. The NNT for the period from randomization to week 1 was determined by analyses of Studies NOH301 and NOH306 (separate and pooled). For the determination of NNT scores from randomization to week 2, separate and pooled results from Studies NOH302 and NOH306 were used. NNT scores from randomization to weeks 4 and 8 were determined using the results from Study NOH306 only.

The NNH with droxidopa was estimated for frequently occurring treatment-emergent AEs (TEAEs) occurring in >5 % of patients treated with droxidopa in Studies NOH301 and NOH302 (pooled data) and/or Study NOH306 based upon TEAEs that occurred in the randomized treatment period. Outcomes of interest for NNH assessment were TEAEs leading to study discontinuation, TEAEs related to falls, and observed supine hypertension (SBP >180 mm Hg at all 3 supine measurements in the orthostatic standing test). The NNH for falls and fall-related injuries assessed in Study NOH306 were calculated separately using falls diary information to determine fall events. Falls information was also collected in Studies NOH301 and NOH302; however, because the falls data were captured retrospectively during these trials, the results are presented only as supportive evidence. Pooled NNHs for discontinuation due to AEs were calculated from all studies with data available at that time point: pooled NNH at week 1 was determined by using data from Study NOH301 and Study NOH306 at week 1, and the pooled NNH at week 2 was determined by using data from Study NOH302 and Study NOH306 at week 2; and NNHs for weeks 4 and 8 were determined by using data from Study NOH306 at those weeks.

The likelihood to be helped or harmed (LHH) was calculated as the ratio of the pooled NNT for the OHSA Item 1 response at weeks 1, 2, 4, and 8 after randomization and the pooled NNH for discontinuations due to TEAEs at the same week.

### Statistics

For each outcome and treatment (droxidopa and placebo), the number of randomized and treated patients and number of patients with an event were determined. The event rates (percentage of patients with an event) by study and for the pooled analysis were calculated for each outcome post hoc, without controlling for multiple analyses; the 95 % CIs were determined using the Wald method [10].

The NNT and NNH values were calculated as reciprocals of the risk difference (difference in event rates) for droxidopa versus placebo. Fractional values were rounded up to the next higher integer. CIs for NNT and NNH were the inverse values of the 95 % CIs for the risk difference if all boundaries were >0; otherwise, the differences were regarded as not statistically significant. LHH was calculated as NNH divided by NNT using values that had not been rounded up to the next integer.

### Ethics, consent, and permissions

All 3 clinical trials received either local or centralized institutional review board approval and were conducted according to the Declaration of Helsinki and its amendments, the International Conference on Harmonisation Good Clinical Practice guidelines, and applicable laws and regulations for each research site. Patients provided written informed consent before study participation.

## Results

### Study population

The most common primary clinical diagnosis was PD in Studies NOH301 and NOH302 (38.8 %–45.1 %); Study NOH306 enrolled only patients with nOH and a primary diagnosis of PD (Table 1) [3, 6]. Patients in Study NOH306 were also slightly older than their counterparts in Studies NOH301 and NOH302. The mean (SD) OHSA Item 1 scores at baseline were similar for the droxidopa and placebo pooled populations (6.0 [2.2] units and 5.8 [2.4] units, respectively) and ranged from 5.1 (2.3) to 6.6 (2.0) units in the individual studies.

**Table 1:** Demographic and baseline characteristics of study populations

Characteristic	Pooled data		NOH301 [3]		NOH302 [6]		NOH306	
	Droxidopa (*n* = 225)	Placebo (*n* = 235)	Droxidopa (*n* = 82)	Placebo (*n* = 80)	Droxidopa (*n* = 50)	Placebo (*n* = 51)	Droxidopa (*n* = 114)	Placebo (*n* = 108)
Mean (SD) age, y	65.0 (14.7)	65.4 (15.6)	57.4 (16.9)	55.7 (20.0)	63.1 (13.8)	66.6 (11.3)	72.6 (7.5)	72.4 (8.0)
Men, *n* (%)	132 (58.7)	142 (60.4)	42 (51.2)	42 (52.5)	30 (60.0)	32 (62.7)	77 (67.5)	68 (63.0)
White, *n* (%)	220 (97.8)	221 (94.0)	82 (100)	75 (93.8)	49 (98.0)	48 (94.1)	110 (96.5)	102 (94.4)
Primary clinical diagnosis, *n* (%)								
PD	150 (66.7)	157 (66.8)	35 (42.7)	31 (38.8)	21 (42.0)	23 (45.1)	114 (100)	108 (100)
MSA	31 (13.8)	25 (10.6)	15 (18.3)	11 (13.8)	17 (34.0)	13 (25.5)	0	0
Pure autonomic failure	34 (15.1)	38 (16.2)	26 (31.7)	28 (35.0)	8 (16.0)	10 (19.6)	0	0
DBHD	0	1 (0.4)	0	0	0	1 (2.0)	0	0
NDAN	4 (1.8)	9 (3.8)	2 (2.4)	6 (7.5)	2 (4.0)	3 (5.9)	0	0
Other	6 (2.7)	5 (2.1)	4 (4.9)^a^	4 (5.0)^b^	2 (4.0)^c^	1 (2.0) ^c^	0	0
OHSA Item 1 score								
*N*	224	236	81	79	50	51	92	105
Mean (SD)	6.0 (2.2)	5.8 (2.4)	6.5 (2.1)	6.2 (2.4)	6.6 (2.0)	6.3 (2.3)	5.4 (2.1)	5.1 (2.3)

*DBHD* dopamine β-hydroxylase deficiency, *MSA* multiple system atrophy, *NDAN* nondiabetic autonomic neuropathy, *OHSA* Orthostatic Hypotension Symptom Assessment, *PD* Parkinson disease

^a^Three patients diagnosed as “likely pure autonomic failure” and 1 patient diagnosed as “likely MSA”

^b^Diagnosed as “likely pure autonomic failure”

^c^Additional information not available

### Number needed to treat

**Fig. 1:**
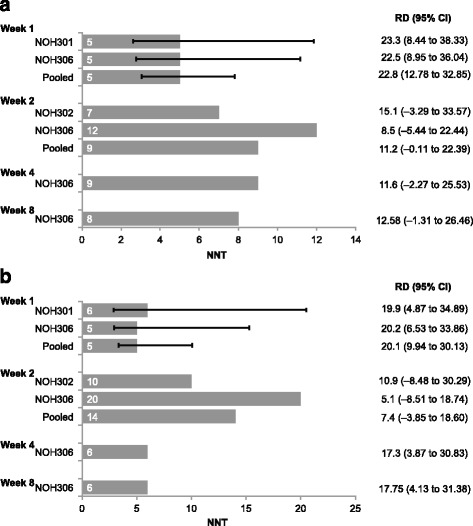
NNT for improvement of ≥2 units (**a**) and ≥50 % (**b**) in OHSA Item 1 (Dizziness/Lightheadedness) Score*. NNT = number needed to treat; OHSA = Orthostatic Hypotension Symptom Assessment; RD = risk difference (between event rates for droxidopa and placebo). *Error bars depicting the 95 % CIs are provided for statistically significant NNT values

A significantly greater percentage of patients treated with droxidopa compared with placebo administration had improvements of ≥2 units or ≥50 % in OHSA Item 1 (dizziness/lightheadedness) scores at week 1 in Study NOH301, Study NOH306, and the pooled analysis (Table 2). The NNT for week 1 with droxidopa to improve OHSA Item 1 score by ≥2 units or by ≥50 % was 5 using pooled data from Studies NOH301 and NOH306 (Fig. 1) and was statistically significant compared with placebo in the pooled analysis. NNT values for week 2 using pooled data from Studies NOH302 and NOH306 were 9 and 14 for improvement of OHSA Item 1 score by ≥2 units and by ≥50 %, respectively. For Study NOH306, the respective NNT values for improvement of OHSA Item 1 score by ≥2 units and by ≥50 % were 9 and 6 at week 4, and 8 and 6 at week 8. The data favored droxidopa at all time points, indicating that droxidopa provided similar efficacy despite differences in patients’ primary clinical diagnoses and mean ages.

**Table 2:** Improvements in OHSA Item 1 scores from randomization to weeks 1, 2, and 4

Improvement, *n* (%)	NOH301			NOH302			NOH306			Pooled		
	Droxidopa (*n* = 82)	Placebo (*n* = 80)	*P* value*	Droxidopa (*n* = 50)	Placebo (*n* = 51)	*P* value*	Droxidopa (*n* = 92)	Placebo (*n* = 105)	*P* value*	Droxidopa (*n* = 174)	Placebo (*n* = 185)	*P* value*
Week 1												
≥ 2 units	53 (64.6)	33 (41.3)	0.004	–	–	–	61 (66.3)	46 (43.8)	0.002	114 (65.5)	79 (42.7)	<0.001
≥ 50 %	45 (54.9)	28 (35.0)	0.017	–	–	–	51 (55.4)	37 (35.2)	0.006	96 (55.2)	65 (35.1)	<0.001
Week 2												
≥ 2 units	–	–	–	36 (72.0)	29 (56.9)	NS	49 (53.3)	47 (44.8)	NS	85 (59.9)	76 (48.7)	NS
≥ 50 %	–	–	–	28 (56.0)	23 (45.1)	NS	38 (41.3)	38 (36.2)	NS	66 (46.5)	61 (39.1)	NS
Week 4												
≥ 2 units	–	–	–	–	–	–	51 (55.4)	46 (43.8)	NS	–	–	–
≥ 50 %	–	–	–	–	–	–	44 (47.8)	32 (30.5)	0.025	–	–	–
Week 8												
≥ 2 units	–	–	–	–	–	–	51 (55.4)	45 (42.9)	NS	–	–	–
≥ 50 %	–	–	–	–	–	–	47 (51.1)	35 (33.3)	0.023	–	–	–

*NS* not significant, *OHSA* Orthostatic Hypotension Symptom Assessment

*P value from the Fisher exact test

### Number needed to harm

#### Frequently occurring adverse events

The most common TEAEs occurring in >5 % of patients treated with droxidopa during the randomized phases of studies NOH301/302 and NOH306 are listed in Table 3. NNH for frequently occurring AEs ranged from 23 to 302 in the pooled analysis, and from -135 to 8647 in any individual study. Only the pooled NNH for hypertension (28; 95 % CI, 15.9–94.6) was significant for droxidopa compared with placebo treatment (Table 3).

**Table 3:** NNH for frequently occurring TEAEs (>5 %) in patients treated with droxidopa during randomized treatment

TEAE	NOH301/302		NOH306		Pooled	
	Droxidopa (*n* = 131)	Placebo (*n* = 132)	Droxidopa (*n* = 114)	Placebo (*n* = 108)	Droxidopa (*n* = 245)	Placebo (*n* = 240)
Headache, *n* (%)	8 (6.1)	4 (3.0)	15 (13.2)	8 (7.4)	23 (9.4)	12 (5.0)
NNH (95 % CI)	33 (NS)		18 (NS)		23 (NS)	
Dizziness, *n* (%)	5 (3.8)	2 (1.5)	11 (9.6)	5 (4.6)	16 (6.5)	7 (2.9)
NNH (95 % CI)	44 (NS)		20 (NS)		28 (NS)	
Nausea, n (%)	2 (1.5)	2 (1.5)	10 (8.8)	5 (4.6)	12 (4.9)	7 (2.9)
NNH (95 % CI)	8647 (NS)		20 (NS)		51 (NS)	
Fatigue, *n* (%)	2 (1.5)	3 (2.3)	8 (7.0)	6 (5.6)	10 (4.1)	9 (3.8)
NNH (95 % CI)	–135 (NS)		69 (NS)		302 (NS)	
Hypertension, n (%)	2 (1.5)	0	8 (7.0)	1 (0.9)	10 (4.1)	1 (0.4)
NNH (95 % CI)	66 (NS)		17 (NS)		28 (15.9–94.6)	

*NNH* number needed to harm, *NS* not significant due to boundaries that include 0, *TEAE* treatment-emergent adverse event

#### Discontinuations due to adverse events

The NNH for AEs leading to discontinuation from the 3 pooled studies was 81, based on overall discontinuation rates due to an AE of 4.49 % (11/245) for droxidopa and 3.25 % (8/246) for placebo. The pooled discontinuation rates due to an AE for droxidopa and placebo (respectively) were 5.64 % (11/195) and 3.08 % (6/195) at week 1 (Studies NOH301 and NOH306), 6.21 % (10/161) and 4.94 % (8/162) at week 2 (Studies NOH302 and NOH306) and 9.0 % (10/111) and 5.4 % (6/111) at both weeks 4 and 8 (Study NOH306 only). NNHs for discontinuations due to AEs were 39 at week 1 (pooled Studies NOH301 and NOH306), 79 at week 2 (pooled Studies NOH302 and NOH306), and 28 at weeks 4 and 8 (Study NOH306).

#### Supine hypertension

Nine patients (7.9 %) treated with droxidopa and 5 patients (4.6 %) receiving placebo experienced supine hypertension in Study NOH306. The NNH for supine hypertension with droxidopa treatment (31) was not significant compared with placebo administration.

#### Falls

Falls and related injuries in Study NOH306B were reported as TEAEs in 15 patients (16.9 %) treated with droxidopa and 21 patients (25.6 %) receiving placebo. The NNH of −12 (12 avoided falls or fall-related injuries during droxidopa treatment) was not statistically significant. Although the data collection in Study NOH301 was done in a different manner (retrospectively), the results from Study NOH306B are supported by Study NOH301 results. In Studies NOH301 and NOH302, falls reported as AEs occurred in 1 patient (0.8 %) treated with droxidopa and in 9 patients (6.8 %) receiving placebo. Using these data, the NNH is −17 (17 avoided falls during droxidopa treatment), but also was not a statistically significant result.

### Likelihood of being helped or harmed

The LHH values calculated from the pooled analysis of the NNT for ≥2 units of improvement in OHSA Item 1 score and the NNH for discontinuations due to AEs were 7.8, 8.8, 3.1, and 3.5 for weeks 1, 2, 4, and 8 after randomization, respectively. For ≥50 % improvement in OHSA Item 1 scores, the calculated LHH values were 7.8, 5.6, 4.7, and 4.7, respectively. Thus, droxidopa is 3.1 to 8.8 times more likely to show a clinical benefit than result in a discontinuation due to an AE compared with placebo.

## Discussion

The key findings of these analyses highlight the efficacy and safety of droxidopa and provide support for the use of improvements in OHSA Item 1 as a benchmark indicator of clinical efficacy in patients with nOH. A significantly greater percentage of patients treated with droxidopa compared with those who received placebo had improvements in OHSA Item 1 scores at week 1. The NNT values were <10 for both OHSA Item 1 improvement of ≥2 units and improvement by ≥50 % at week 2 in the pooled study population and at weeks 4 and 8 in Study NOH306. The similarity of these results is indicative of the overall efficacy of droxidopa, regardless of the individual studies analyzed.

A small decrease in droxidopa response rates over time was noted, although the overall variability at different time points (the effects were less evident at week 8 than weeks 2 or 4) suggests that these observations are possibly trial artifacts and are not supported by longitudinal trial data in Study NOH306. It is unclear why this effect occurs, but there could be at least 3 potential contributors to this effect. First, results are pooled from 3 studies with varying enrollment/inclusion criteria, differing study designs, and outcome measurements. Although this heterogeneity does not create a concern for pooling data at any single time point, the pooling of different studies at different time points may result in challenges when results are examined longitudinally. Of note, Study NOH306 contributes data at each time point examined (ie, weeks 1, 2, 4, and 8), but Study NOH301 contributes data only at week 1, and Study NOH302 only contributes data at week 2. Secondly, it has been suggested this finding could be related to physiologic factors (eg, adaptation of adrenoreceptors in response to droxidopa therapy [5]), or psychometric factors (eg, response shift, a phenomenon associated with patient-reported outcomes in which positive treatment effects influence the patient’s recall of baseline symptoms [11]). Finally, the observed response trends may be a result of increased adherence to nonpharmacologic nOH management techniques within the placebo group that were encouraged in both arms as part of the clinical trial protocols [5].

With regard to the safety of droxidopa, the NNH of the pooled studies for AEs leading to study discontinuation was high (NNH = 81). For specific AEs, the NNHs were also high, with only the NNH for hypertension being significantly different between droxidopa and placebo. The LHH for droxidopa was favorable, being 8.6 times more likely to show a clinical benefit than result in AEs that would lead to discontinuation of treatment. In our study, supine hypertension was defined as SBP >180 mm Hg. Use of this threshold underestimates supine hypertension if the condition is defined by a lower SBP value. However, it should be noted that patients with nOH experience blood pressure variability and supine hypertension as part of the natural course of the disease [12]. Because patients with nOH have a fundamental inability to control their blood pressure and may often experience blood pressures >160 mm Hg when supine (without treatment), thresholds for hypertension applicable to the general population may not be relevant in a nOH population.

In 2 of the studies (NOH301 and NOH302) included in our analyses, only patients who met the responder definition during the open-label dose optimization period and continued to the double-blind treatment period were included. Although pooling data across the 3 studies provides a more robust estimate of NNT and NNH for droxidopa treatment, the post hoc analyses of the trial results introduce methodological challenges. The method for calculating NNT and NNH requires a comparison group; thus, only the post-randomization period results from Study NOH301 and NOH302 could be included in the analyses. This led to the exclusion of the 181 patients who were not treatment responders during the open-label titration optimization in these 2 trials; therefore, there could be a concern that the NNT analyses might be biased in favor of droxidopa. The magnitude of the bias can be examined by comparison of the results in Table 2 from Studies NOH301/NOH302 (in which only open-label responders were randomized) to Study NOH306 (in which all patients were randomized). At week 1, the absolute risk reduction for ≥2 units of OHSA Item 1 improvement between Study NOH301 (23.3 %) and Study NOH306 (22.5 %) are similar (with analogous results found for the ≥50 % improvement). At week 2, there is a considerable difference between absolute risk reduction for ≥2 units of improvement in OHSA Item 1 in Study NOH302 and Study NOH306 (15.1 % vs 8.5 %); however, the week 2 results in Study NOH306 are somewhat anomalous when compared to outcomes at other weeks in the study. At weeks 1, 4, and 8 (excluding week 2), the absolute risk differences for ≥2 units of improvement in OHSA Item 1 found in Study NOH306 were 22.5 % (as noted above), 11.6 %, and 12.5 %, respectively (Table 2). Thus, because the results at these other weeks are similar, it is clear that the risk difference at week 2 (8.5 %) is an outlier that makes it seem that the pooled Study NOH301/302 results are biased in favor of droxidopa. Overall, it appears that any bias in the NNT resulting from the study design/randomization strategy in Studies NOH301 and NOH302 is not large, regardless of different study designs in Study NOH302 (open-label titration period followed by randomized treatment withdrawal) and NOH306 (all patients were randomized).

As noted above, estimation of the NNH also requires a control group to compare the incidence of AEs versus the treated group. Thus, it is not possible to determine the NNH impact of AEs in the open-label period of Studies NOH301/NOH302. In Study NOH301, 12 patients discontinued participation due to an AE during the open-label period, and in Study NOH302, there were 43 patients who discontinued the study prior to randomization due to an AE/blood pressure elevation, resulting in a total of 12.4 % (55/444) patients not completing the open-label titration phase due to an AE (including blood pressure elevation). In Study NOH301, the most frequently reported AEs leading to discontinuation were nausea (1.5 %), or hypertension (0.8 %) [3]. In Study NOH302, the most frequently reported AE leading to discontinuation was dizziness (*n* = 3) [6]. During the open-label periods of Studies NOH301 and NOH302, the AE profile for all patients was qualitatively similar to that during randomized treatment; the most frequently reported AEs were headache, dizziness, nausea, fatigue, and falls. Although use of only randomized patient data may provide less conservative treatment effect estimates for a patient initiating droxidopa in routine clinical practice, it does not appear that inclusion of patient data from the open-label period of NOH301 and NOH302 would substantially impact the NNT/NNH interpretation or the clinical significance of the results.

Dizziness/lightheadedness is the cardinal symptom of nOH [1, 7], and improvement of this symptom may represent the most clinically relevant benefit of nOH treatment for patients. The OHQ was developed to assess the severity and impact of symptoms in patients with nOH [7]. The OHQ was initially validated in patients with nOH [7] and more recently proved to be an effective tool for assessing the severity of orthostatic hypotension in older patients (median age, 67) with a clinical diagnosis of orthostatic hypotension [13]. However, benchmarks for clinically relevant improvements in the symptoms of nOH have not been fully evaluated. The consistency of NNT values between using an improvement of ≥2 units or ≥50 % in OHSA Item 1 scores and the fact that NNT values were <10 suggest that the use of these criteria/endpoints to assess the efficacy of droxidopa for the treatment of nOH are clinically meaningful [9]. The clinical relevance of droxidopa may be further examined by the NNH for falls in Study NOH306B where falls were a secondary endpoint, recorded through patient-reported electronic diary entries, with fall injuries determined from prespecified AE types (occurring on the day of or day after a fall) [5]. The result suggests that droxidopa treatment is associated with a greater number of avoided falls and fall-related injuries compared with placebo.

A potential limitation of this analysis for NNT determination is that there is currently no gold standard for demonstrating a treatment response for patients with nOH. However, using 2 definitions of improvement in OHSA Item 1 (≥2 units of improvement or ≥50 % improvement), similar NNT values were obtained, suggesting that these endpoints may be good indicators of a clinically meaningful response in patients with nOH. A second potential limitation is that using discontinuations due to AEs as a clinically relevant proxy for overall tolerability to determine NNH may lead to a conservative estimate of NNH because of the assumption that all AEs were due to study drug. It should be pointed out that the pooled calculated NNH for discontinuations due to AEs (81) is within the range of NNHs based on individual AEs (from 23 for headache to 302 for fatigue in the pooled analysis). A further limitation of these analyses is that for the assessment of LHH, the selection of outcomes used for NNT and NNH determination can be subjective as their relative importance to the patient may vary [9]. As discussed above, the endpoints chosen for NNT and NNH determinations yielded consistent results with respect to the efficacy and safety endpoints.

## Conclusions

Droxidopa is an efficacious drug for the treatment of nOH, with NNTs below 10 following weeks 1, 2, 4, and 8 of treatment and an acceptable tolerability profile, with NNH values ranging from 20 to 8647 for specific TEAEs. Furthermore, based on the LHH for the pooled analysis at week 1, compared with placebo, droxidopa is 7.8 times more likely to show a clinical benefit than result in a discontinuation due to an AE. In addition, when the analysis is limited to injuries due to falls, there appears to be a consistent numerical trend for decreased injuries favoring droxidopa.

## Abbreviations

AE: Adverse Event
DBHD: Dopamine β-hydroxylase Deficiency
DBP: Diastolic Blood Pressure
FDA: US Food and Drug Administration
LHH: Likelihood of Being Helped or Harmed
MSA: Multiple System Atrophy
NDAN: Nondiabetic Autonomic Neuropathy
NNH: Number Needed to Harm
NNT: Number Needed to Treat
nOH: Neurogenic Orthostatic Hypotension
NS: Not Significant
OHDAS: Orthostatic Hypotension Daily Activity Scale
OHQ: Orthostatic Hypotension Questionnaire
OHSA: Orthostatic Hypotension Symptom Assessment
PD: Parkinson Disease
SBP: Systolic Blood Pressure
TEAE: Treatment-emergent Adverse Event
TID: 3 Times Daily
